# Impact of Nitrogen Sources on Gene Expression and Toxin Production in the Diazotroph *Cylindrospermopsis raciborskii* CS-505 and Non-Diazotroph *Raphidiopsis brookii* D9

**DOI:** 10.3390/toxins6061896

**Published:** 2014-06-20

**Authors:** Karina Stucken, Uwe John, Allan Cembella, Katia Soto-Liebe, Mónica Vásquez

**Affiliations:** 1 Alfred Wegener Institute Helmholtz Centre for Polar and Marine Research, Am Handelshafen 12, 27570 Bremerhaven, Germany; E-Mails: kstuckenm@gmail.com (K.S.); Uwe.John@awi.de (U.J.); Allan.Cembella@awi.de (A.C.); 2 Department of Molecular Genetics and Microbiology, Faculty of Biological Sciences, Pontificia Universidad Católica de Chile, Alameda 340, Santiago, Chile; E-Mail: katiasotoliebe@gmail.com; 3 Millenium Nucleus EMBA, Alameda 340, Santiago, Chile

**Keywords:** cyanobacteria, *Cylindrospermopsis*, *Raphidiopsis*, cylindrospermopsin, saxitoxin, nitrogen, gene expression

## Abstract

Different environmental nitrogen sources play selective roles in the development of cyanobacterial blooms and noxious effects are often exacerbated when toxic cyanobacteria are dominant. *Cylindrospermopsis raciborskii* CS-505 (heterocystous, nitrogen fixing) and *Raphidiopsis brookii* D9 (non-N_2_ fixing) produce the nitrogenous toxins cylindrospermopsin (CYN) and paralytic shellfish toxins (PSTs), respectively. These toxin groups are biosynthesized constitutively by two independent putative gene clusters, whose flanking genes are target for nitrogen (N) regulation. It is not yet known how or if toxin biosynthetic genes are regulated, particularly by N-source dependency. Here we show that binding boxes for NtcA, the master regulator of N metabolism, are located within both gene clusters as potential regulators of toxin biosynthesis. Quantification of intra- and extracellular toxin content in cultures at early stages of growth under nitrate, ammonium, urea and N-free media showed that N-sources influence neither CYN nor PST production. However, CYN and PST profiles were altered under N-free medium resulting in a decrease in the predicted precursor toxins (doCYN and STX, respectively). Reduced STX amounts were also observed under growth in ammonium. Quantification of toxin biosynthesis and transport gene transcripts revealed a constitutive transcription under all tested N-sources. Our data support the hypothesis that PSTs and CYN are constitutive metabolites whose biosynthesis is correlated to cyanobacterial growth rather than directly to specific environmental conditions. Overall, the constant biosynthesis of toxins and expression of the putative toxin-biosynthesis genes supports the usage of qPCR probes in water quality monitoring of toxic cyanobacteria.

## 1. Introduction

Cyanobacterial blooms often dominate the photosynthetic plankton in eutrophic freshwater and coastal brackish waters. Numerous forms of nitrogen (N), primarily nitrate/nitrite, ammonium, urea and cyanate can be assimilated by cyanobacteria [1]. Additionally, many cyanobacteria fix dinitrogen (N_2_), and therefore can survive and even thrive in N-depleted environments. Cyanobacteria produce a wide array of toxic secondary metabolites (cyanotoxins) [2,3], among them, the hepatotoxic microcystins and nodularins, the cytotoxic cylindrospermopsins and apratoxins, the dermatotoxins lyngbyatoxins, and the neurotoxic anatoxins. Putative gene clusters have been identified for the biosynthesis of these toxins; all encode for large multienzymatic complexes of non-ribosomal peptide synthetases (NRPS), polyketide synthases (PKS) or by hybrid NRPS/PKS complexes [4,5,6,7,8,9]. The paralytic shellfish poisoning toxins (PSTs) are neurotoxins produced in freshwater by cyanobacteria and in marine environments by dinoflagellates [10]. The putative biosynthesis pathway of PSTs is directed by a novel PKS and additional non-NRPS/PKS enzymes [11].

Independent of the biosynthesis pathway, all cyanotoxin chemical structures fall into cyclic peptides or alkaloids, thus the majority are highly nitrogenated. The availability and chemical composition of N sources are believed to influence cyanobacterial blooms, specifically in the succession of N_2_-fixing and non N_2_-fixing species [12]. These two aspects lead to several studies on the relationship between N-input and toxicity in microalgae, particularly in microcystin producers. In N-limited batch and continuous cultures of *Microcystis aeruginosa*, the specific microcystin production rate correlated to cell division rate at favorable cell growth conditions [13,14]. Induction of growth by nitrate, however, did not induce changes to microcystin cell quotas and consequently toxin genes (*mcy*) were not upregulated [15]. In *Planktothrix agardhii*, amino acid supplementation affected the ratio of microcystins toxic variants; however, a shift from the N-saturated to N-limited medium only produced a decrease in the total toxin content correlated with a decrease in cell biomass and not a shift in the toxin variants as expected [16]. 

N availability also plays a pivotal role in dinoflagellate blooms [17,18], and it is believed to influence PST production [19,20]. However, when normalized to cell growth, the studies indicate that under N-limitation, dinoflagellates divide slower while increasing their cell size, thereby accumulating toxins (increase in cell quota) instead of producing more [20]. Contrary to this, in the diazotroph *Aphanizomenon* *flos-aquae*, N-limitation increased PST production at the end of the growth phase [21]. However, in a diazotrophic organism such as *A. flos-aquae*, N-limitation is rapidly overcome by N_2_ fixation and implications of N-limitation at later stages of growth cannot be inferred.

Studies on cylindrospermopsin production and release have been carried out in a few cyanobacterial species, *Cylindrospermopsis raciborskii*, *Aphanizomenon* spp. and *Oscillatoria.* Modulation of N-sources (ammonium and N-deprivation) in *C. raciborskii* showed that higher toxin concentrations were produced under N-deprivation [22]. Toxin measurements were only performed in exponential phase, and gene expression data were not available at that time. In a different study, *A. ovalisporum* N-starvation resulted in a reduction of the cellular CYN content while transcript levels of the CYN biosynthetic genes, *aoaA* and *aoaC*, decreased independently of the amount of toxin recorded [23]. Other abiotic factors such as temperature, light and phosphate concentration have shown to affect CYN production and release [24,25,26,27,28,29], but toxin gene expression or protein activity was reported only in one study [27]. 

Understanding the factors that affect cyanotoxin production would provide a tool to monitor, predict, and prevent the emergence of toxic blooms of cyanobacteria, and with the previous identification of the toxin biosynthesis genes, quantification of these in blooms is now possible. Given the critical importance of N supply and metabolism in growth, bloom development and life history transitions of cyanobacteria, we aimed our study to compare the effects of alternative nitrogen sources in the cyanobacterial production of cylindrospermopsins and PSTs and their molecular regulation. The filamentous diazotroph *Cylindrospermopsis raciborskii* which produces CYN and its analog, deoxycylindrospermopsin (doCYN) [30], in addition to the non-diazotrophic *Raphidiopsis brookii* which produces gonyautoxins GTX2/3 and their respective decarbamoyl analogs, dcSTX and dcGTX2/3 [31], were used as model organisms*.* From both strains the genomes and putative toxin biosynthesis gene clusters have previously been elucidated [8,32,33]. These are located adjacent to genes regulated by NtcA, the master regulator for N availability raising the hypothesis that toxin synthesis could be regulated by nitrogen in these two cyanobacteria. We explored the relationship between toxin production and transcriptional regulation of *ntcA* and of the main putative genes for toxin biosynthesis and transport in *C. raciborskii* CS-505 and *R. brookii* D9. 

## 2. Results

### 2.1. Growth and Toxin Production under Different N-Regimes

**Figure 1:**
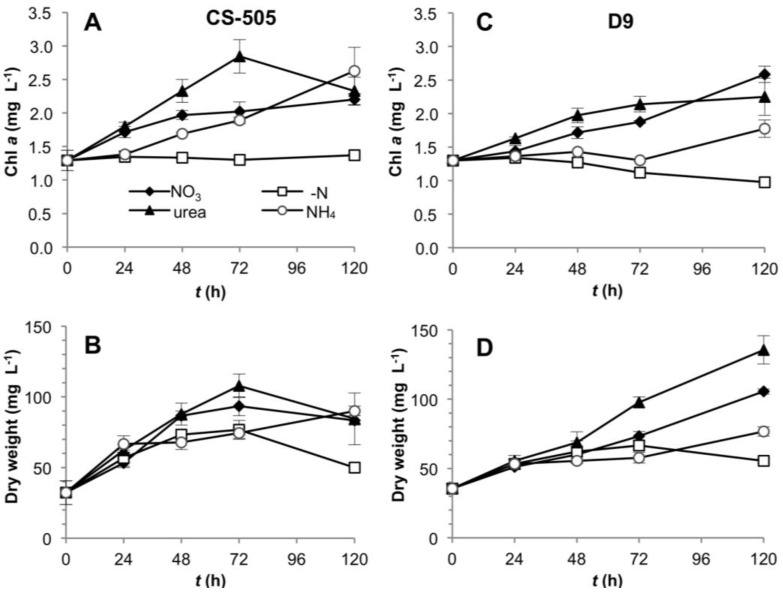
Growth curves of CS-505 (**A**,**B**) and D9 (**C**,**D**). Curves were plotted by chlorophyll *a* and dry weight per unit culture volume. Values are shown as the average of three biological replicates; error bars indicate ±SD from the mean (*n* = 3).

The growth and toxin production of *C. raciborskii* CS-505 and *R. brookii* D9 (heretofore called CS-505 and D9, respectively) were studied under nitrate, ammonium or urea as substrate or under the absence of dissolved N (Figure 1). Experiments were performed from cultures adapted to grow on nitrate in a five-day period to observe the early response to N-deprivation of CS-505 and D9. This response will be followed by N_2_ fixation and cell death on the respective strains. Growth of both strains was not apparently dependent on the N-source at the time studied, with the exception of the lower biomass yield in CS-505 under N-deprivation (Figure 1B), and the growth lag and consequently lower biomass of D9 under ammonium growth (Figure 1D). Besides the expected N-deprivation-induced cell bleaching, chl *a* measurements were in agreement with dry weight biomass estimations (Figure 1A,C) and OD_750_ (Table S1). 

**Figure 2:**
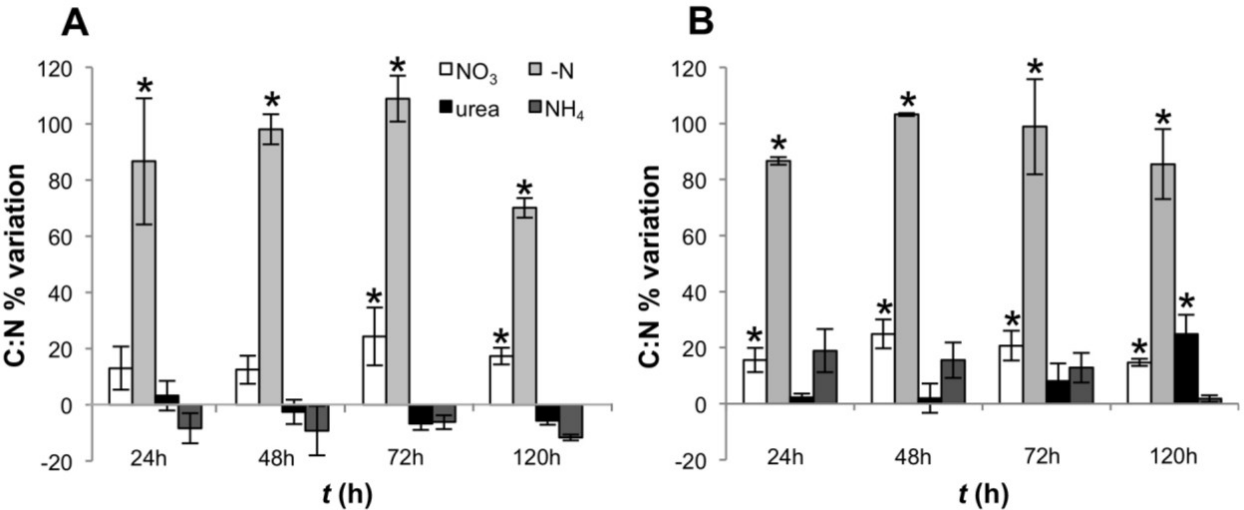
C:N ratios of CS-505 (**A**) and D9 (**B**) grown under alternative N-regimes. Values are shown as a percentage of variation with respect to *t* = 0; error bars indicate ±SD from the mean (*n* = 3). Values significantly different from *t* = 0 (one way ANOVA, Tukey’s HSD *post hoc* test *p* < 0.01) are marked with a * symbol.

The decrease in the intracellular levels of N, as reflected by high C:N ratios in cultures growing in N-free medium, was apparent after 24 h and remained high during the entire experiment (Figure 2). However, a significant decrease in the C:N ratio was observed after 120 h in CS-505 (one way ANOVA, Tukey’s HSD *post hoc* test *p* < 0.01) as reflected by an increase in the intracellular nitrogen pool while particulate carbon (PC) remained constant (Figure 2A and Figure S1).

**Figure 3:**
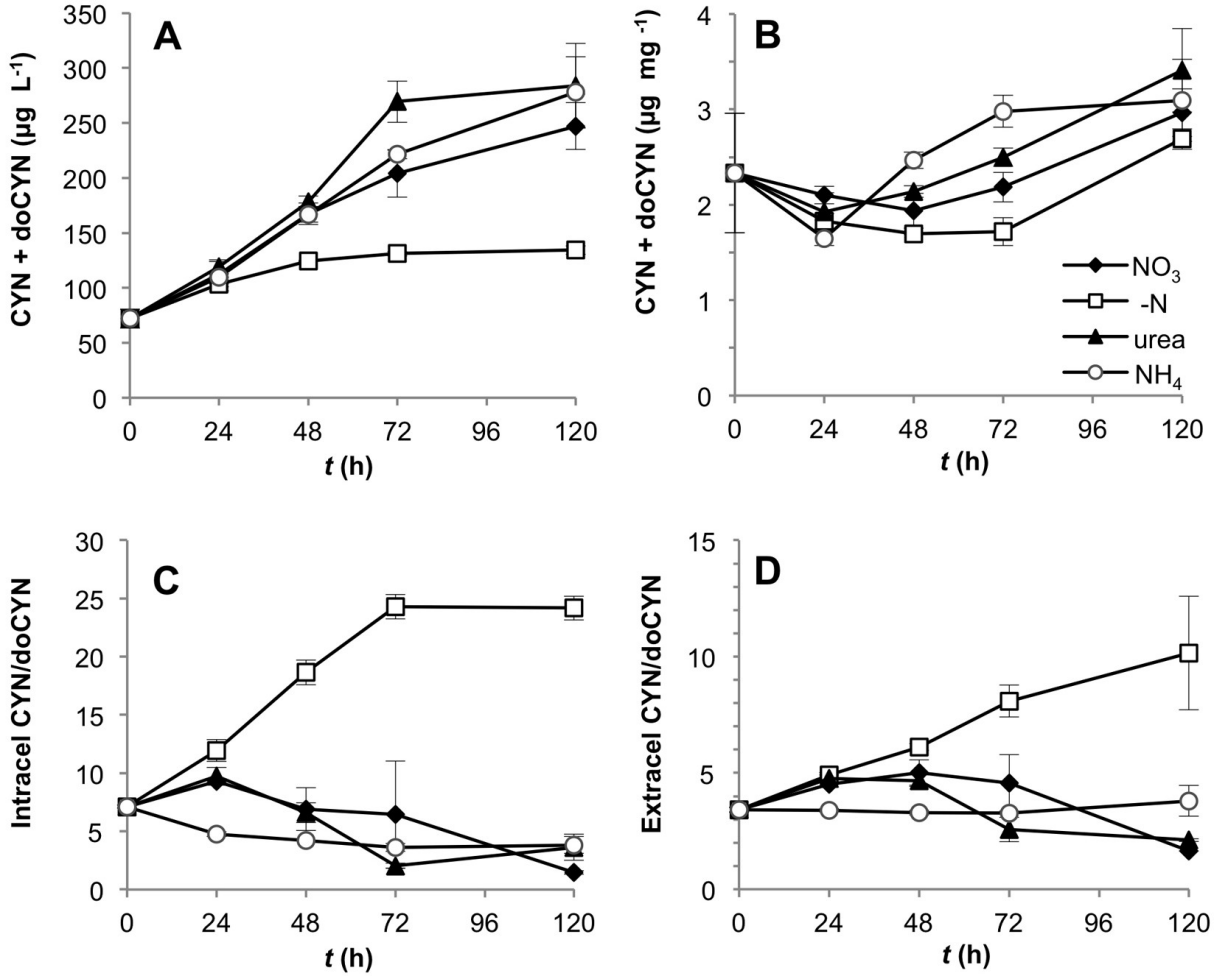
Toxin production by CS-505 grown under four alternative N-regimes. (**A**) Total CYN + doCYN content over the time in the intra- and extracellular component per unit culture volume; (**B**) total toxin content normalized to biomass (dry weight); (**C**) and (**D**) intra- and extracellular ratios of CYN:doCYN, respectively.

Total intra- and extracellular CYN and doCYN concentration increased with time in CS-505 cultures under all N-treatments (Figure 3A). This response was, however, much lower in the treatment without dissolved N. When normalized to biomass, the effect of N-removal on toxin production correlated with the available N pool in the cells (Figure S1); the toxin content per unit biomass remained low until 72 h, then increased until 120 h (Figure 3B), along with a drop of almost 50% in the C:N ratio (Figure 2A). These changes reflect an increase in cellular N, probably derived from N_2_ fixation. Similarly, N-deprivation significantly affected the intra- and extracellular CYN: doCYN, ratio (one way ANOVA, Tukey’s HSD *post hoc* test *p* < 0.01), which increased as reflected by a dramatic decrease in the precursor doCYN (Figure 3C,D). The CYN:doCYN ratios progressively decreased in the cellular and extracellular fraction under all fixed N-sources (Figure 3C,D), although this trend was not as marked by 120 h in the extracellular fraction (Figure 3D). Small differences in this tendency are likely attributable to cell lysis and/or leakage of toxins rather than to active toxin transport.

**Figure 4:**
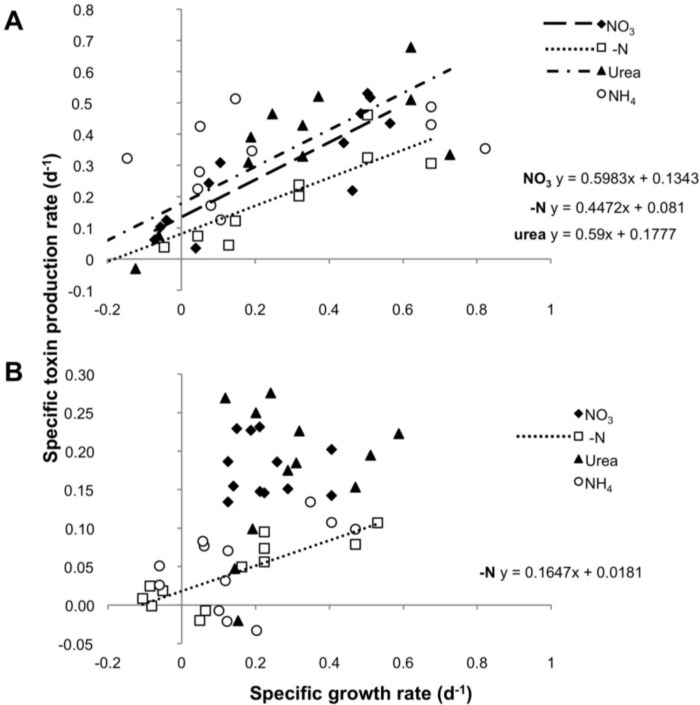
Specific toxin production rate as function of specific growth rate in the four N regimes for CS-505 (**A**) and D9 (**B**).

To test a possible correlation between toxin production and cell division, for each strain we compared the rates of toxin production (µ*_tox_*) and growth rate (µ*_c_*) in all four experimental settings. µ*_tox_* and µ*_c_* showed a significant correlation in cultures grown on nitrate and urea over the entire growth curve, with a slope not significantly different from 1 (ANCOVA: *p* = 0.204550 for nitrate and *p* = 0.566347 for urea), suggesting a balanced equilibrium between net growth and toxin production. In contrast, cultures grown on N-free medium showed a correlation between µ*_tox_* and µ*_c_* whose slope (*m* = 0.447) differed significantly from a slope = 1 (ANCOVA: *p* = 0.000930). No significant correlation was observed for the ammonium treatment, for which the toxin production rate appeared to be independent of growth rates (Figure 4A).

**Figure 5:**
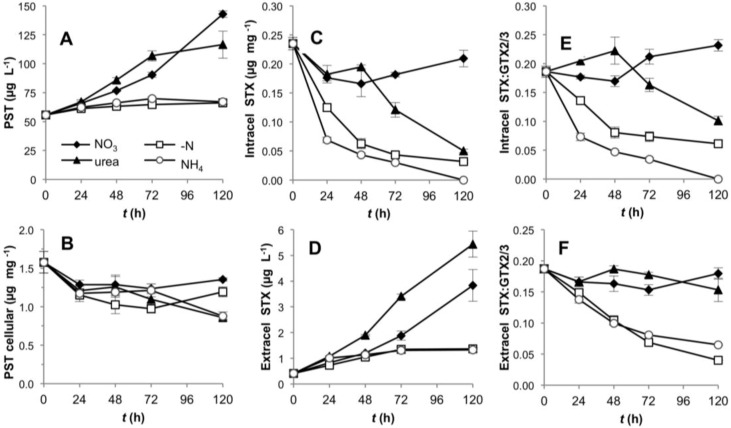
Toxin production by D9 grown under four alternative N-regimes. (**A**) Total PST content over the time in the intra- and extracellular component per unit culture volume; (**B**) total toxin content normalized to biomass (dry weight); (**C**) and (**D**) intra- and extracellular STX content; (**E**) and (**F**) intra- and extracellular STX:GTX2/3 ratios.

*R. brookii* D9 produces six PST analogs in the following order of descending relative abundance: GTX2/3 > STX > dcSTX and dcGTX2/3. We explored by HPLC the production of all six analogs and represented our values as total toxin content, and ratios between STX and the epimers GTX2/3. The total PST content (intra- and extracellular) of *R. brookii* D9 grown on nitrate or urea increased along the time course of growth, but remained roughly constant in cultures grown with ammonium or under N-deprivation over the 120 h experiment (Figure 5A). The total toxin per unit biomass (µg·PSTs·mg^−1^) decreased during the first 24 h in all N-treatments, and continued to decrease in the ammonium and urea treatments, where it reached a minimum of 0.86 µg·mg^−1^ (approx. 50% of the initial amount) at 120 h (Figure 5B). In contrast, under -N conditions and with nitrate, the total PST content per unit biomass increased towards the end of the experiment but did not attain the initial levels. The increase of total PSTs in the -N treatment may be explained by the low biomass at this time point while the total PSTs remained constant (relatively refractory to degradation) in the medium. The trend towards decreasing intracellular toxins, except for the nitrate treatment, was only reflected in the increase in the extracellular STX pools in cultures grown on urea (Figure 5C,D). 

In the intracellular fraction, the toxin composition, expressed as the STX:GTX2/3 ratio, rapidly shifted down in the -N and ammonium treatments, while decreasing only later in the urea-grown cultures (Figure 5E). In the ammonium treatment, the intracellular content of STX decreased to the limit of detection (Figure 5C), thereby producing lower STX: GTX2/3 ratios; the same effect was observed in the -N treatment (Figure 5E,F). In these cases, the cultures were in two different physiological stages; N-depleted cultures were destined to die from starvation, whereas cultures grown on ammonium showed a lag phase only until the fourth day, after which they increased in biomass and chlorophyll *a* content (Figure 1). This lag phase suggests acclimation of the cells for a different assimilation strategy of dissolved N. With nitrate as growth substrate, the STX: GTX2/3 ratio (approximately 1:5) did not significantly change (one way ANOVA, Tukey’s HSD *post hoc* test *p* < 0.01) over the first 48 h, but increased slightly in the latter half of the experiment (Figure 5E,F). In general, the ratios in the intracellular and extracellular fractions followed similar patterns, with the exception of the urea treatment, which showed a downshift only in the intracellular component, presumably due to the release of STX to the extracellular medium. At this stage we cannot assess if this is an active or passive toxin release.

The rate of PST production was apparently independent of the growth rate in the urea, nitrate and ammonium treatments. Only the -N treatment showed a significant and positive correlation between these two parameters. Nevertheless, the slope of the regression line (*m* = 0.1647) was significantly different from a slope = 1 (ANCOVA *p* = 0.000116), showing that under N deprivation, a doubling in the biomass did not yield a corresponding doubling in toxin content (Figure 4B). 

### 2.2. NtcA Binding Boxes and Transcriptional Regulation of cyr and sxt Genes

By computational predictions we inferred the presence of transcriptional units (TUs) within the cylindrospermopsin (*cyr*) and saxitoxin (*sxt*) gene clusters based on the assumption of gene proximity (*i.e.*, intergenic region <50 nt) and the lack of regulatory boxes inside the intergenic regions. We could allocate two TUs, *cyrDFGI* and *cyrABE*, in the *cyr* cluster of CS-505 (Figure S2A) and five TUs, *sxtQRST*, *sxtLSUL*, *sxtIJ*, *sxtFGH*, and *sxtABC*, in the *sxt* cluster of D9 (Figure S3A). NtcA motifs were predicted as described above (see experimental section). The presence of a −10 box with the motif TAN_3_T, which appears downstream of all known NtcA binding sites [34,35] was considered as essential. Under these criteria, NtcA binding motifs were predicted for the *hypF* (part of the hydrogenase gene cluster, N-regulated in cyanobacteria), *cyrABE*, *cyrJ*, and *cyrK* genes (Figure S2B-E). Flanking the *sxt* gene cluster is the glutamine synthetase gene *glnA*, a known target for NtcA regulation [36]. Aside from the putative NtcA binding region for *glnA* (Figure S3B), NtcA motifs were predicted upstream of the *sxtM* and *sxtABC* genes (Figure S3C,D). In addition, other four motifs were found inside the coding sequences of the *sxtA*, *sxtP*, *sxtQ*, and *sxtS* genes (Figure S3D,E).

Candidate genes for the biosynthesis of the toxin precursor molecules (STX or CYN), tailoring reactions, toxin transport, and predicted functions are shown in Table 1. The expression of four *cyr* and eight *sxt* genes in cultures grown in nitrate, ammonium or urea were compared with N-free medium, considering nitrate-dependent growth as a control under which CYN and PSTs are synthesized constitutively. Thus, transcript abundance is expressed as the percentage of variation respect to time 0 (cells grown on nitrate) from absolute quantification values, which are obtained from standard curves and do not depend on normalization by a housekeeping gene (see experimental section). qRT-PCR values for D9 on N-free medium were not obtained because the filaments did not grow under this condition and produced only poor quality RNA after 24 h. 

The expression of the N-regulator gene *ntcA* was determined as a proxy control for N-regulation (Table 1). With ammonium, the levels of *ntcA* showed a decrease after 72 h in CS-505. In D9, the mRNA levels of *ntcA* continuously decreased until 72 h in cells grown on nitrate and ammonium, as we expected for the negative regulation of *ntcA* under N-replete conditions. In the urea treatment, *ntcA* showed a two-fold up regulation at 72 h in CS-505, whereas the D9 expression remained unchanged. The *ntcA* transcript levels increased three-fold only after 48 h of N-deprivation, yet the C:N ratios were high after the first 24 h. 

Cylindrospermopsin and saxitoxin biosynthetic genes were constitutively expressed under all treatments tested (Table 1), but toxin production was slower during the 120 h of the experiment in the -N treatment (Figure 3 and Figure 4). Under N-deprivation, the *cyrB* transcript level dropped 65% after the first 24 h; after 48 h, the levels increased but remained below the initial values until the end of the experiment. The *cyrI* and *cyrJ* transcripts decreased at a lesser extent (35%) at 24 h, but they returned to the initial levels and decreased at 120 h, whereas *cyrK* transcripts did not change. In N-replete treatments, the gene expression response was rather surprising. After 120 h, the transcript levels did not rise above the control, with the exception of *cyrB* at 48 h in ammonium, and at 72 h in nitrate, and *cyrJ* at 48 h in urea. Gene expression in the ammonium and nitrate treatments showed a similar response, namely a downregulation between 72–120 h. With urea, *cyrI* and *cyrK* were repressed at 24 and 48 h. The eight STX biosynthetic genes assayed responded similarly and comparably to the *cyr* genes; a tendency to downregulation was observed near the end of the time series (Table 1). 

**Table 1:** Transcript abundance of *ntcA*, *cyr* and *sxt* genes under alternative N-sources.

Gene/Predicted Function/Reference	NtcA Motif	Time (h)	% Transcript Relative to Time 0			
			NO_3_	NH_4_	urea	-N
*ntcA* **(CS-505)** Transcriptional regulator in nitrogen metabolism [37]	Y	24	60.3 ± 3.9	89 ± 7.5	132.3 ± 15.4	110.5 ± 12.7
		48	135.9 ± 22	139.1 ± 4.8	220.7 ± 20	283.1 ± 17.6
		72	107.2 ± 16.3	80.6 ± 12.8	NM	155.9 ± 14.9
		120	136.2 ± 20.1	47.8 ± 9.1	NM	181.8 ± 7.6
*ntcA* **(D9)** Transcriptional regulator in nitrogen metabolism [37]	Y	24	63.5 ± 6.8	98.7 ± 8.7	87.8 ± 13.3	NM
		48	54.2 ± 9.5	56.3 ± 2.8	111 ± 15.7	NM
		72	16.6 ± 5.8	27.4 ± 1.3	NM	NM
		120	49 ± 1	30.5 ± 5.1	NM	NM
*cyrB* NRPS/PKS, second step in CYN biosynthesis [8]	Y	24	62.8 ± 1.7	93 ± 21	121.9 ± 25.2	35.1 ± 9
		48	107.4 ± 4.7	141.8 ± 15.9	124.2 ± 58.7	75.3 ± 7.4
		72	126.3 ± 3.2	73 ± 4.6	NM	64.1 ± 8.9
		120	20.1 ± 5	29.8 ± 8.9	NM	65.4 ± 2.1
*cyrI* Hydroxylation of C-7 in doCYN to form CYN [8,38]	N	24	73.8 ± 8.8	80 ±7.8	121.9 ± 25.2	35.1 ± 9
		48	96.4 ± 18.6	141.8 ± 15.9	124.2 ± 58.7	75.3 ± 7.4
		72	110.3 ± 11.7	73 ± 4.6	NM	64.1 ± 8.9
		120	66.7 ± 16	29.8 ± 8.9	NM	65.4 ± 2.1
*cyrJ* Sulfotransferase [8]	Y	24	74.4 ± 6.1	74.2 ± 3.3	106.2 ± 18.5	66.4 ± 10.2
		48	107.2 ± 1.6	114.9 ± 1.7	179 ± 21	89.9 ± 12.3
		72	43.5 ± 3	76.7 ± 6.6	NM	60.7 ± 17.3
		120	54 ± 9.5	31.9 ± 9.8	NM	64.1 ± 9.4
*cyrK* Transport of CYN/doCYN [8]	Y	24	93.2 ± 4.6	68.3 ± 5.6	52.4 ± 1.8	106.3 ± 15.9
		48	101 ± 0.4	96.9 ± 13.5	63.6 ±12.2	114.9 ± 10
		72	44.5 ± 5.9	60.9 ± 1.7	NM	86.6 ± 11.6
		120	38.1 ± 7.3	29.2 ± 9.9	NM	96.7 ± 13.4
*sxtSUL* Sulfotransferase rendering GTX2/3 [39]	N	24	38.5 ± 8.4	160.2 ± 5.7	61.3 ± 13	NM
		48	36.2 ± 9.4	131.4 ± 21.7	98.4 ± 31.4	NM
		72	20.5 ± 1.2	47.2 ± 4.8	NM	NM
		120	19.5 ± 3	46.2 ± 6	NM	NM
*sxtDIOX* Hydroxylation of STX prior to GTX2/3 formation [39]	N	24	85.8 ± 7.6	116.1 ± 25	57 ± 3.2	NM
		48	146 ± 11.4	104.9 ± 20.3	78 ± 7	NM
		72	74.5 ± 6.4	51.8 ± 4.7	NM	NM
		120	71.6 ± 1.3	75.5 ± 1.2	NM	NM
*sxtM* PST export [11]	Y	24	83.8 ± 11.7	161.3 ± 18.2	69.2 ± 6.4	NM
		48	60.8 ± 2.8	141 ± 25.5	122.5 ± 25	NM
		72	45.11 ± 4.4	71.9 ± 5.1	NM	NM
		120	48.1 ± 3.9	80 ± 6.6	NM	NM
*sxtF* PST export [11]	N	24	84.5 ± 13	152.3 ± 14.3	86.8 ± 2.9	NM
		48	64.9 ± 3.6	83 ± 14.1	86.9 ± 5.5	NM
		72	52.4 ± 3	72.6 ± 11	NM	NM
		120	65.9 ± 6.2	70.5 ± 6	NM	NM
*sxtA* ACP/aminotransferase, first step in STX biosynthesis [11]	Y	24	116.2 ± 10.5	165.8 ± 10.6	124 ± 3.7	NM
		48	77.5 ± 6.5	97 ± 10.4	160.7 ± 26	NM
		72	67.6 ± 2	36.6 ± 2.2	NM	NM
		120	58.2 ± 4.3	30.6 ± 1.2	NM	NM
*sxtO* Donor of sulfate group [11]	N	24	90.4 ± 3.8	60.3 ± 0.8	36 ± 6.9	NM
		48	60.6 ± 3.9	51.4 ± 4.2	57.5 ± 8.5	NM
		72	30.1 ± 2.9	22 ± 3.6	NM	NM
		120	35.6 ± 7.8	15.3 ± 1.5	NM	NM
*sxtI* Carbamoyltransferase [11]	N	24	47.3 ± 4.2	179.1 ± 8.8	86.5 ± 8.1	NM
		48	51.9 ± 3.1	106.9 ± 10.7	104.9 ± 9.5	NM
		72	35.5 ± 4.1	59.1 ± 5.7	NM	NM
		120	39.5 ± 5.7	49.1 ± 8.4	NM	NM
*sxtU* Reduction of C-1, eighth step in STX biosynthesis [11]	N	24	61.8 ± 8.2	125.8 ± 17.3	91.4 ± 13.5	NM
		48	62.2 ± 2.8	123.4 ± 21.2	132.3 ± 13.4	NM
		72	40.2 ± 0.3	82.1 ± 5.6	NM	NM
		120	37.6 ± 4.8	53.2 ± 1.7	NM	NM

Transcript abundance was calculated from absolute quantification measurements. Samples were taken from three biological replicates at 24 h intervals during 120 h. Urea samples were taken only until 48 h. Standard deviation of the mean (*n* = 3) is shown. Y: Yes; N: No; NM: not measured. In bold and underlined is shown the value where transcript levels surpassed more than two-fold that of nitrate growth.

## 3. Discussion

This work represents an integrated assessment of the physiological and transcriptional responses of two filamentous toxigenic cyanobacteria to growth on alternative N regimes. Both *C. raciborskii* CS-505 and *R. brookii* D9 demonstrated the capacity to grow on a variety of fixed N-sources. Independent of the N-regime, cells committed to cell division upon transfer from nitrate stock medium completed division, but under N-deprivation, further growth was arrested in both CS-505 and D9. Removal of nitrate from the culture medium caused a decrease in the intracellular N pools leading to an increased C:N ratio as early as 24 h in both species, indicating that the cells were under nitrogen limitation (Figure 2 and Figure S1). However, a significant decrease in the C:N ratio was observed after 120 h in CS-505 (one way ANOVA, *p* < 0.01) as reflected by an increase in the intracellular nitrogen pool while particulate carbon (PC) remained constant (Figure 2A and Figure S1). *C. raciborskii* CS-505 increased, likely as a consequence of N_2_ fixation and the time required for heterocyst development [40]. 

In *C. raciborskii* CS-505 (SDS), the highest concentrations of CYN have been previously recorded at late stationary phase in cultures grown at a moderate photon flux density of 140 μmol m^−2^·m^−1^ [41] and in the absence of a dissolved N-source [22]; the lowest concentrations were found in several *C. raciborskii* strains when cultures were supplemented with ammonium [22]. In *Aphanizomenon* spp*.* and *C. raciborskii*, a decrease of CYN accumulation was also reported as a result of increased temperature [25,26,28]. Our results highly contrast with Saker and Neilan’s findings [22], but in their report, CYN concentrations were recorded at the end of the exponential growth phase where *C. raciborskii* cultures have restored the N-status via N_2_ fixation and therefore the cells are no longer N-depleted. 

Our study aimed to detect the early response towards changes in nitrogen availability excluding the acclimation of the cultures. Within this time frame, CS-505 and D9 showed constitutive toxin biosynthesis in all treatments where N was supplied as a dissolved source. This is in agreement with reports of CYNs and PST production in laboratory strains [24,26,42,43,44] and of CYNs in natural environments [45]. Based on the conditions tested in this study, the general implication is that the effect of the N-source is not directly related with toxin production in CS-505 and D9, but rather conditions that negatively affect growth also affect toxin production, as seen under N-deprivation and ammonium. Our results show a clear positive correlation of toxin production to cell growth under nitrate and urea growth for CS-505 (Figure 4A), both treatments which promoted growth. Similar results were shown earlier in *C. raciborskii* by Hawkins *et al.* [44] where CYN production correlated with growth rate at exponential phase, in *A. ovalisporum* [29], and are well described for microcystin producers [13,14]. However, they are in contradiction with reports on *Aphanizomenon* spp. and *C. raciborskii* where CYN quotas decoupled from growth rates [24,25,26].

The transcriptional regulator NtcA binds to the consensus sequence GTAN_8_TAC located at the promoter region of its target genes [36,46]. Upon N limitation, NtcA activates the genes responsible for uptake and assimilation of alternative nitrogen sources, although it can also act as a repressor [1]. Our results showed that even when cells had reduced levels of particulate intracellular nitrogen after 24 h (Figure S1), induction of *ntcA* was observed by qRT-PCR only after 48 h, after which the transcript levels remained high. If the target genes were regulated by NtcA, transcript abundance should have increased (or decreased) after 48 h of N removal; however, for most of the eight *sxt* and four *cyr* gene transcripts quantified by qRT-PCR, there was no transcriptional regulation for the period studied. This suggests that under the studied conditions at early growth stage, STX and CYN biosynthesis is not regulated at the transcriptomic level. 

Studies in *A. ovalisporum* have shown that starvation for sulfate and phosphorous (P) produced a reduction of the cellular CYN content [29] whereas N starvation did not affect CYN content and increasing light intensity resulted in a larger accumulation of CYN in the cells and in the medium [23]. Transcript levels of the CYN biosynthetic genes, *aoaA* and *aoaC* (equivalent to *cyrA* and *cyrC* of *C. raciborskii*) decreased in the high light and N starvation experiments indicating that toxin production is regulated by additional mechanisms. Our results support this proposition since removal of N from the medium did not cause changes in the toxin content. An ArbB regulator was found to bind the upstream region of the *aoaA* and *aoaC* genes in *A. ovalisporum*, but no further experiments were carried on to demonstrate the involvement of this transcriptional regulator on CYN biosynthesis [23].

In our cultures, CS-505 cells growing on atmospheric N showed an increased CYN:doCYN ratio. In CYN biosynthesis, doCYN is the precursor that is hydroxylated by the enzyme CyrI to form CYN in a 2-oxoglutarate (2-OG)-dependent manner [38]. Since total CYN and doCYN content was constant, one possible explanation for the increased CYN:doCYN ratio would be an accelerated rate of conversion from doCYN to CYN given by the increased 2-OG levels under N deprivation [47]. CyrI activity assays under various concentrations of 2-OG are required to test this hypothesis. Recent reports showed that the proportion of doCYN to CYN in *C. raciborskii* is strain dependent and suggested that the changes in the ratio could be given by changes in *cyrI* activity or variation of the gene sequence [42,48]. Our qRT-PCR data showed no expression changes of *cyrI*, and only a slight downregulation of *cyrB*, involved in the synthesis of the precursor doCYN, supporting the first hypothesis. 

The variation of PST composition observed in cultures grown with ammonium and under atmospheric N might be related to the stability of the protein complexes that synthesize STX and its analogs. Currently, the post-translational regulation (if any) of the proteins in this pathway remains unknown. Only two studies, with contradictory concluding remarks, reported the effect of chloramphenicol (CAM), a protein synthesis inhibitor, on PST biosynthesis in *C. raciborskii* T3 and *R. brookii* D9 (initially misclassified as *C. raciborskii* D9). Whereas Pomati *et al.* [49] suggested that PST biosynthetic enzymes have a high turnover rate (based on 76% inhibition of STX with CAM after 24 h), Soto *et al.* [31] found an early accumulation of STX (but not of GTX2/3), but they could not measure intracellular toxins after 24 h because CAM, in the applied concentrations, induced total cell lysis. The decrease of STX in D9 cultures grown on ammonium (down to the analytical limit of detection) and under N-deprivation, points towards a high turnover rate of STX biosynthetic enzymes. 

A decrease of STX:GTX2/3 ratios was observed in ammonium and N-deprivation, in which cells showed slow growth. Thus, a direct correlation of PST analog inter-conversion with N-status (2-OG levels) cannot be inferred as with CYN:doCYN. The inter-conversion of STX into GTX2/3 under low growth must be studied at the enzymatic level. Shifts in the toxin variants have been reported for microcystins, in which increasing nitrogen availability leads to an increase of the nitrogen-rich variant microcystin-RR [50]. 

The cosmopolitan distribution of these toxic cyanobacterial species and their constitutive production of potent toxins pose increasing challenges for predicting and managing the consequences to aquatic ecosystems and human health. Furthermore, notwithstanding several efforts to identify the ecological and evolutionary functions of cyanobacterial toxins, this aspect remains elusive. It was proposed that in *Aphanizomenon ovalisporum*, CYN promotes its own inorganic phosphate supply by inducing alkaline phosphatase secretion by other phytoplankton [27]; similar effects were reported when *M. aeruginosa* was treated with purified CYN and with extracellular medium from a non-toxic *C. raciborskii* strain [51]. Despite the increase in alkaline phosphatase activity in *M. aeruginosa*, this effect was not exclusive for CYN and the authors concluded that *C. raciborskii* produces a hitherto unknown metabolite with allelopathic properties that mimics the effect of CYN. Furthermore, since *C. raciborskii* has a high affinity for phosphate [52], a role of CYN on phosphate acquisition seems unlikely. Accumulating evidence suggests that PSTs could act extracellularly as a protective mechanism to ensure homeostasis against extreme salt variation in the environment [39,49]. As now, the role of CYN as and PSTs in *C. raciborskii* and *R. brookii* (if any) remains to be elucidated. The results presented here show that both cyanobacterial toxin types, CYNs and PSTs, are constitutively synthesized in a growth-dependent manner, independently of the nitrogen source. This implies a role other than N-metabolism. 

## 4. Experimental Section

### 4.1. Cultures and Experimental Settings

*Cylindrospermopsis raciborskii* strain CS-505 was isolated from the Solomon Dam in Palm Island, Queensland, Australia and obtained from the CSIRO culture collection, Hobart, Australia. *Raphidiopsis brookii* D9 (originally classified as *C. raciborskii* D9) is a clonal isolate from the Billings freshwater reservoir near Sao Paulo, Brazil. Batch cultures of non-axenic *C. raciborskii* CS-505 and *R. brookii* D9 were grown in MLA medium on 2 mM sodium nitrate [53] in a controlled environmental chamber on a 12/12 h light/dark photocycle at a photon flux density of 35 µmol·m^−2^·s^−1^ at 25 °C. All experiments were performed in three biological replicates starting from a stock culture. To change the experimental N regime, 800 mL of stock culture in early exponential growth phase (OD_750_ of 0.2) were filtered through an 8 µm pore-size cellulose ester membrane (Millipore, Darmstadt, Germany) which serves as a filter for removal of contaminating free-living bacteria. Cells were resuspended in twice the volume of new MLA medium containing 2 mM sodium nitrate, 2 mM ammonium chloride, 1 mM urea, or without a dissolved external N-source but with 2 mM NaCl to restore osmotic balance. In order to allow for faster growth, experiments were performed for 120 h under a continuous light regime at a photon flux density described above.

For determination of biomass, chlorophyll *a*, C:N ratios, toxins and RNA isolation, samples from each of the three biological replicates were harvested at time points of 0, 24, 48, 72 and 120 h after the exchange of the N-source. Estimation of biomass was based on optical density (OD_750_) of 3 mL cell culture and on dry weight from 30 mL harvested cells. Chlorophyll *a* was extracted from 1 mL samples in technical replicates and measured according to the ISO method [54]. For C:N ratios, technical duplicates (4 mL) from each biological replicate were filtered through a prebaked (500 °C, 5 h) GF/F filter (GE Healthcare, Freiburg, Germany) and analyzed with an Elemental Analyzer (Euro EA, HEKAtech, Wegberg, Germany). 

Cellular CYN and PSTs were harvested from 30 mL of experimental cultures after centrifugation (20 min at 3220 *g*) at room temperature and pellets were frozen at −20 °C until extracted. For the extracellular toxin fraction, 10 mL of supernatant after centrifugation were filtered through a 0.2 μm pore-size nitrocellulose membrane (Carl Roth, Karlsruhe, Germany). To avoid degradation of PSTs, immediately after filtration the pH of the supernatant was adjusted to 2.5 with HCl and the extract was stored at −20 °C.

Frozen cell pellets and supernatants were lyophilized (Beta I Freeze Dryer, Martin Christ, Germany) at −20 °C and *ca*. 0.004 mbar vacuum. Intra- and extracellular toxins were extracted in 500 μL and 300 μL of 0.05 M acetic acid, respectively. Samples were disrupted with an ultrasonic cell disruptor (Sonoplus Bandelin Electronics, Berlin, Germany) for 1 min; extracellular toxins were transferred to vials and stored at −20 °C until analyzed. Intracellular toxin extracts were centrifuged at 5000 *g* for 10 min at 4 °C and filtered through a 0.45 μm pore-size membrane filter (Millipore, Darmstadt, Germany) and stored at −20 °C until analysis. 

### 4.2. Analysis of Toxins

PSTs were determined by high performance liquid chromatography with fluorescence detection (LC-FD) following post-column oxidation. Details of the LC-FD analysis, equipment and PST standards are as previously described [55]. 

Cylindrospermopsin (CYN) and deoxycylindrospermopsin (doCYN) were identified and quantified by liquid chromatography coupled to tandem mass spectrometry (LC-MS/MS). Mass spectral experiments were performed on an ABI-SCIEX-2000 triple quadrupole mass spectrometer (Applied Biosystems, Darmstadt, Germany) coupled to a Model 1100 liquid chromatographic system (Agilent, Waldbronn, Germany). 

Separation of CYN and doCYN was performed by reverse-phase chromatography on an analytical column (150 × 3 mm) packed with 3 µm Luna C18 (2) 120 Å (Phenomenex, Aschaffenburg, Germany) and maintained at 20 °C. The flow rate was 0.3 mL·min^−1^ and gradient elution was performed with two eluents, where eluent A was water and eluent B was methanol/water (95:5 v/v), both containing 2.0 mM ammonium formate and 50 mM formic acid. Initial conditions were 10 min column equilibration with 10% B, followed by a linear gradient to 90% B in 15 min and isocratic elution until 19 min with 90% B. The system was then returned to initial conditions until 20 min (total run time: 30 min). 

Selected reaction monitoring (SRM) experiments were carried out in positive ion mode by selecting the following transitions (precursor ion > fragment ion): *m/z* 400 > 194 and *m/z* 400 > 176 for doCYN and *m/z* 416 > 194 and *m/z* 416 > 176 for CYN. Dwell times of 100 ms were used for each transition. Sample concentrations were calibrated against external standards of CYN and doCYN.

Specific growth and toxin production rates (µ*_c_* and µ*_tox_*, respectively) for both PSTs and CYN/doCYN were calculated for each interval in the growth and toxin production curves using either cell biomass (dry weight) or toxin concentration values according to Anderson *et al.* [56]. Both specific rates are reported in units of d^−1^.

### 4.3. Statistical Analysis

All experiments were conducted with three independent biological replicates and results are presented as the mean value (±SD). The means were evaluated with one way ANOVA for multiple comparisons among groups, followed by *post hoc* comparison using Tukey’s HSD. 

### 4.4. RNA Isolation

Cells for RNA isolation were collected by filtration of 50 mL of culture through an 8 µm pore-size cellulose ester membrane (Millipore, Darmstadt, Germany). Filtered cells were resuspended in 800 µL of RLT lysis buffer (Qiagen, Hilden, Germany), and flash frozen in liquid nitrogen, in a complete process of less than 2 min. Samples were stored at −70 °C and total RNA was extracted with the RNeasy mini-kit (Qiagen, Hilden, Germany) following the manufacturer’s instructions. Briefly, frozen samples were thawed on ice, and cells were disrupted twice for 2 min with 0.1 mm diameter acid-washed glass beads in a tissue lyser (Qiagen, Hilden, Germany). The supernatant was separated from the glass beads and cell debris by centrifugation (10 min, 16,000 *g*, 4 °C). DNA digestion was performed after RNA isolation for 1 h at RT, followed by a final clean-up with a second 30 min DNA on-column digestion. The presence of genomic DNA in the RNA samples was tested by PCR. RNA was quantified by spectrophotometry (ND-1000, NanoDrop Technologies, Wilmington, DE, USA) and purity was assessed based upon the ratios A_260_/A_230_ and A_260_/A_280_. If required, samples were further cleaned with a Microcon Elute System (Millipore, Germany). RNA integrity was checked on RNA nano-chips using an Agilent Bioanalyzer 2100 (Agilent Technologies, Waldbronn, Germany). 

### 4.5. qRT-PCR Conditions and Motif Search

The cDNA for qRT-PCR was synthesized from 500 ng total RNA using random hexamers with the Omniscript RT kit (Qiagen, Hilden, Germany). The qRT-PCR reactions were performed in 20 µL reaction mixtures composed of 1 µL 10-fold diluted cDNA, forward and reverse primers at a concentration of 100 nM, and 10 µL 2× SYBR Green PCR Master Mix (Applied Biosystems, Darmstadt, Germany). Cycle parameters were as follows: initial denaturation at 95 °C (10 min), followed by 40 cycles of 95 °C (15 s) and 59 °C (1 min). Finally, a product-primer dissociation step was utilized to verify formation of a single unique product/primer dimerization.

All qRT-PCR primers were designed with Primer Express 3.0 software (Applied Biosystems, Darmstadt, Germany) and synthesized by Eurofins MWG Operon (Ebersberg, Germany). Samples were run in biological and technical triplicates, and mean values were taken among these replicates, including standard deviation. For each primer pair, a standard curve was established by 10-fold dilutions of a PCR template, spanning concentration differences from 100 pg to 1 fg. Linear regression analysis between PCR product concentration and the cycle number (Ct-value) was used to determine primer efficiency and for absolute quantification of each gene transcript. The qRT-PCR amplicon sizes, primer sequences and efficiencies are shown in Table S2. For absolute quantification, cDNA values were obtained from Ct values using the linear regression from the standard curve (Table S2). Copy numbers of each gene transcript were calculated based on the molar mass and length derived from the qRT-PCR amplicon sequences using the Avogadro constant as previously described [57]. Copy numbers were normalized to total RNA of the sample to obtain the absolute copy number of gene transcripts/ng total RNA. Transcript abundance was expressed as the percentage of variation respect to time = 0 (cells grown in nitrate).

The Motif Search tool from the Nano+Bio-Center at the University of Kaiserslautern [58] was employed to search for putative NtcA-binding domains in the intergenic regions outside and within the *cyr* (41.6 Kb) and *sxt* (25.7 Kb) gene clusters. The NtcA-binding domains have a canonical sequence defined as GTAN_8_TAC, and they are separated by approximately 22 nucleotides from a −10 box with the consensus sequence TAN_3_T [34]. However, differences in the canonical structure and in the length of separation between the promoter and binding site have been described and probed for genes such as *petH*, *nifH* and *cphA1* [34]. Following these criteria, we set the mismatches to 1, to allow variation in the six conserved positions of the NtcA-binding site. The results were further filtered according to the presence of a −10 box with the conserved motif TAN_3_T for NtcA-biding regions.

## 5. Conclusions

In *C. raciborskii* and *R. brookii*, both CYNs and PSTs, respectively, are constitutively produced along the growth curve and, to the best of our knowledge, there are no conditions that could arrest toxin production whilst growth conditions are positive. Here we showed that N-deprivation and cytotoxicity caused by ammonia (only in D9), negatively affect CYN and STX biosynthesis in *C. raciborskii* CS-505 and *R. brookii* D9, respectively, but in neither case is there evidence that these toxins are transcriptionally regulated as classic inducible/repressible secondary metabolites in response to environmental stress factors. In fact, their production dynamics and regulatory features are more typical of components of primary or intermediary metabolism, albeit of unknown function. In any case, the shifts in the ratios of toxin analogs with a clear independency of biosynthetic gene regulation strongly indicate that shifts in enzymatic activity or post-transcriptional mechanisms are involved in toxin biosynthesis and regulation.

## Supplementary Files

Supplementary File 1 Supporting Information (PDF, 1149 KB)
