# Supplementary material for: Impact of Nitrogen Sources on Gene Expression and Toxin Production in the Diazotroph Cylindrospermopsis raciborskii CS-505 and Non-Diazotroph Raphidiopsis brookii D9
Source: Toxins (Basel). 2014 Jun 20;6(6):1896–915. doi: 10.3390/toxins6061896 (PMC4073136; doi:10.3390/toxins6061896)
Supplement: Supplementary File 1 — Supporting Information (PDF, 1149 KB) [file toxins-06-01896-s001.pdf]

Supporting Information

Figure S1. Variation of particulate nitrogen and carbon of CS-505 and D9 under alternative N regimes.

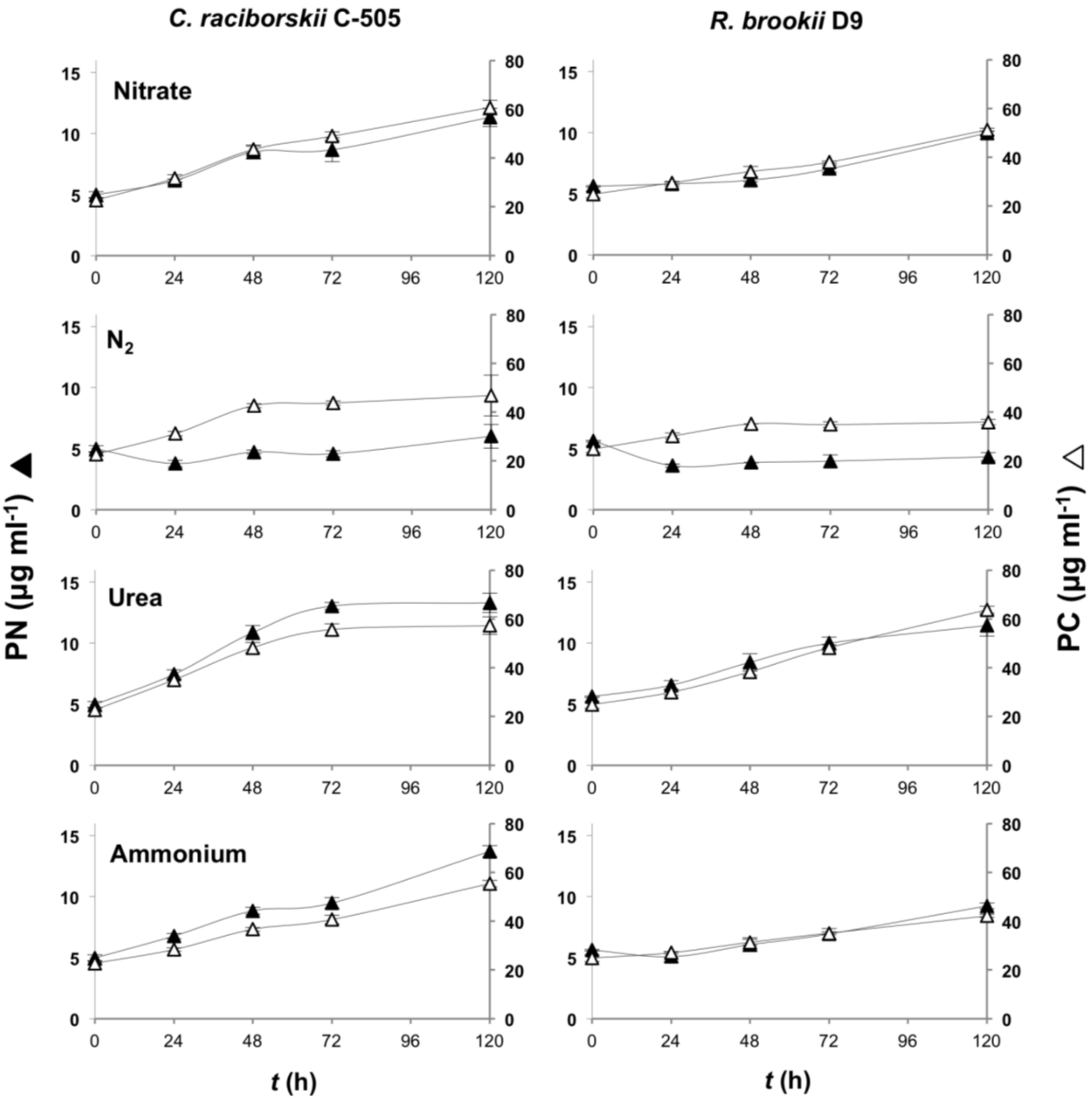

Figure S2. *In silico* analysis of transcriptional units (TU), NtcA binding boxes and promoters outside and within the *cyr* gene cluster. (**A**) Graphical representation of the *cyr* gene cluster and predicted TUs. Genes forming part of TUs are marked by black lines across them. (**B**–**E**) Nucleotide sequence of regions containing predicted NtcA binding boxes. NtcA binding boxes are shown as a thick red line within the cluster and enclosed in rectangles in the sequences. The −10 and −35 regulatory boxes are shown only for the −10 box with the conserved motif TAN_3T.

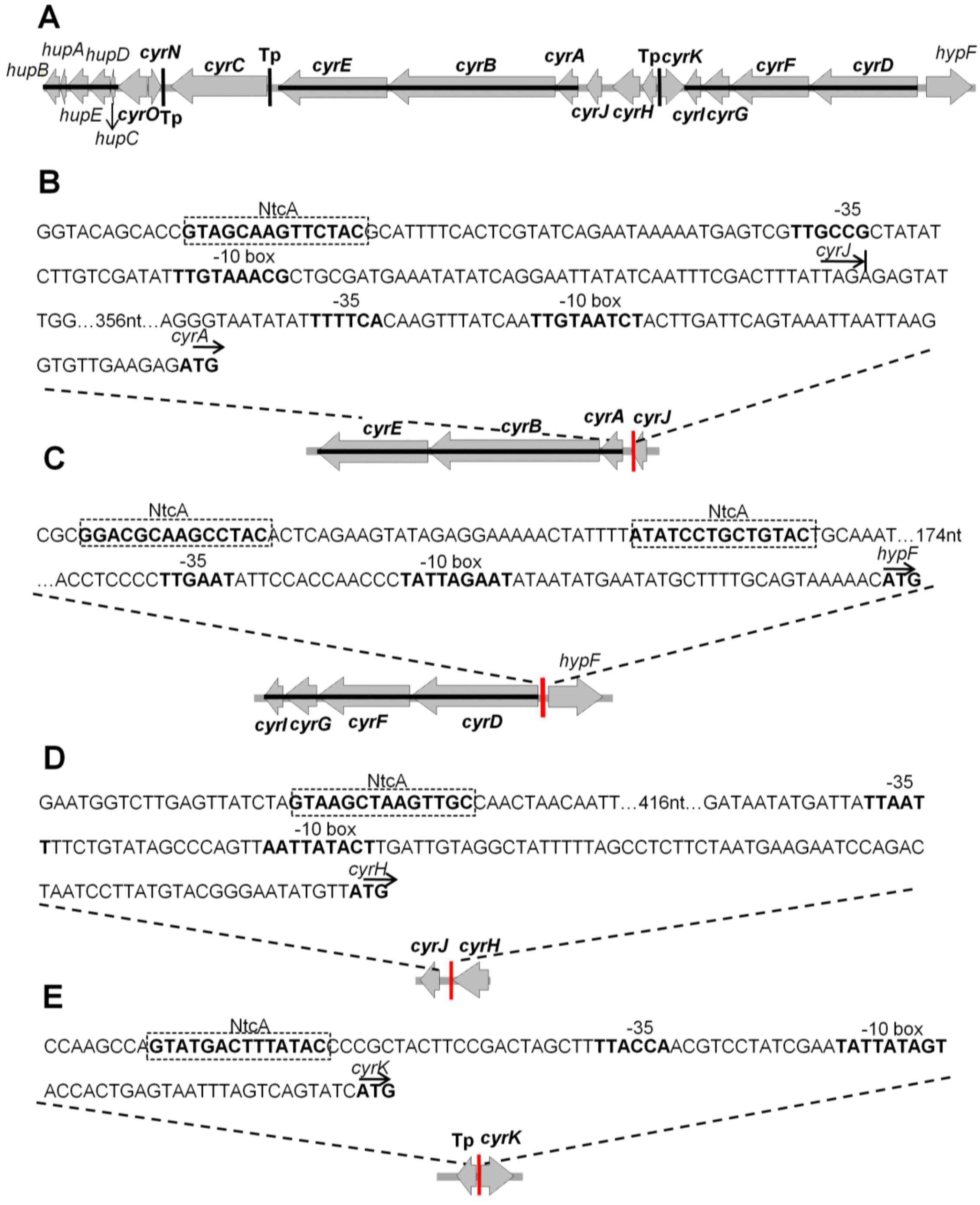

Figure S3. *In silico* analysis of transcriptional units (TU), NtcA binding boxes and promoters outside and within the *sxt* gene cluster. (**A**) Graphical representation of the *sxt* gene cluster and predicted TUs. Genes forming part of TUs are marked by black lines across them. (**B**–**E**) Nucleotide sequence of regions containing predicted NtcA binding boxes. NtcA binding boxes are shown as a thick red line within the cluster and enclosed in rectangles in the sequences. The −10 and −35 regulatory boxes are shown only for the −10 box with the conserved motif TAN_3T. In the presence of two possible −10 and −35 boxes within the same intergenic region, the second sequence appears underlined.

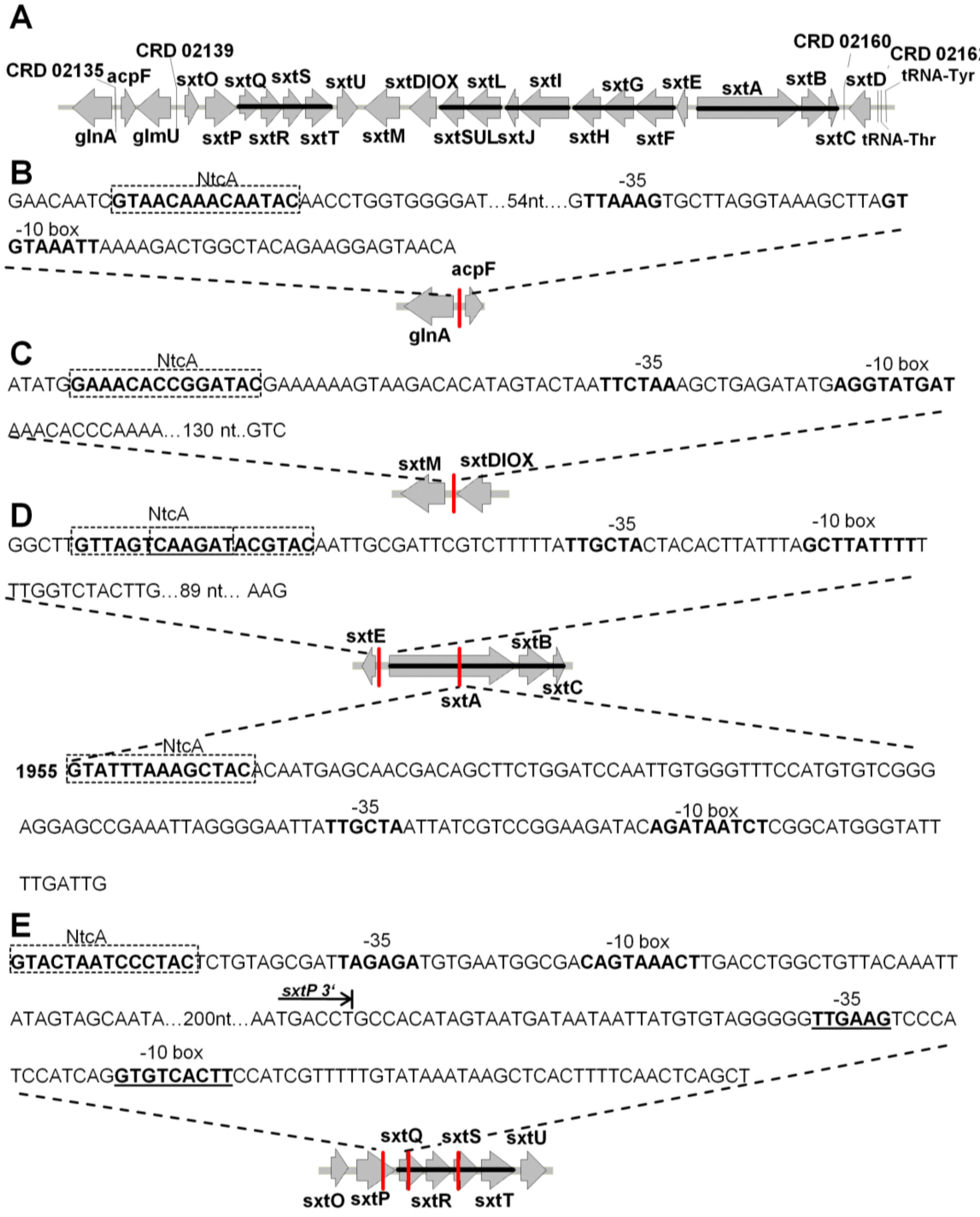

Table S1. Pearson correlation coefficients ($p < 0.001$) between biomass estimations based on chlorophyll *a* (chla), optical density (OD) and dry weight (DW).

Biomass estimation	Pearson Corr (r) *C. raciborskii* CS-505				Pearson Corr (r) *R. brookii* D9			
	Nitrate	N2	Urea	Ammonium	Nitrate	N2	Urea	Ammonium
chla *vs*. DW	0.90	0.14	0.95	0.79	0.97	−0.35	0.90	0.82
chla *vs*. OD	0.97	0.25	0.95	0.91	0.95	−0.25	0.88	0.48
OD *vs*. DW	0.93	0.81	0.97	0.90	0.97	0.95	0.99	0.80

Values in green boxes have $p > 0.001$.

Table S2. qPCR primer sequences, efficiencies and amplicon sizes.

Gene	Amplicon Size (nt)	qRT-PCR Primer Sequence 5′-3′	Slope	Efficiency (%)
ntcA	60	qD9ntcAF TCGCTGAAGCAATAGGATCCA<br>qD9ntcAR TCCCGCAAGTCTCCTAGCAA	−3.33	100.3
	60	q505ntcAF TTTTACTGCGGTGGAATTGCT<br>q505ntcAR TTCCTTGAGGGCCTGCTCTAC	−3.41	102.7
sxtSUL	79	qsxt14F CTGAAGTAGCGGCTCAACAGATAA<br>qsxt14R CAGGAGGATTGCGAAGCATAA	−3.67	110.5
sxtDIOX	99	qsxt15F CGATGGGAGAAATTGCGAAT<br>qsxt15R ATGTCAGGGTGAGCTGGGATA	−3.76	113.1
sxtO	69	qsxtOF TTGGGTGAGGTTGCCAAACT<br>qsxtOR CGGTCATTCCTGTAGGGTGAGA	−3.53	106.3
sxtU	69	qsxtUF GCGATCGCCGCAAGAC<br>qsxtUR CTTGACCACCACTGGCTTCA	−3.31	99.7
sxtA	59	qACPR GGGAGAGCGAGCCTTGAAT<br>qACPR ATGCGGGACAACATAGGAGTGT	−3.30	99.0
sxtI	69	qsxtIF TTGTTGCTGCAGCTCAGGAA<br>qsxtIR ATCGCTCCAGTCGGAAACC	−3.29	99.1
sxtM	100	qsxtMF GGCTAGCAACGGCCTTGTC<br>qsxtMR TGCCAGTAACACATTGCTTTGTT	−3.16	95.2
sxtF	60	qsxtFF GCCCATGATATTGGCTTCCA<br>qsxtFR TGCCGACTCCGAGTGGTATAAC	−3.47	104.5
cyrB	99	qcyrBF ACTGCACAGACTGCGATTTCA<br>qcyrBR TGCGCCCCATGTTAATGTATT	−3.48	104.8
cyrI	94	qcyrIF TCCCGGTCATCCATCAGTAAG<br>qcyrIR ATGTGGGTGCGTCTTCTTGATA	−3.65	109.9
cyrJ	60	qcyrJF TGAACGATCCGCGGAGAA<br>qcyrJR TCGGTAAACTCAACCCCTACAAC	−3.32	100.0
cyrK	99	qcyrKF AGATGACCGAGAGGGCGTACT<br>qcyrKR CACCCCTAACGGGTACTGTAACA	−3.97	119.5

© 2014 by the authors; licensee MDPI, Basel, Switzerland. This article is an open access article distributed under the terms and conditions of the Creative Commons Attribution license (http://creativecommons.org/licenses/by/3.0/).
